# Effectiveness of neuromuscular electrical stimulation combined with rehabilitation training for treatment of post-stroke limb spasticity

**DOI:** 10.1097/MD.0000000000017261

**Published:** 2019-09-27

**Authors:** Ya-long He, Yan Gao, Bai-ya Fan

**Affiliations:** Department of Neurology, Yan’an People's Hospital, Yanan, China.

**Keywords:** effectiveness, limb spasticity, neuromuscular electrical stimulation, post-stroke, rehabilitation training, safety

## Abstract

**Background:**

This study aims to systematically explore the effectiveness of neuromuscular electrical stimulation (NMES) combined with rehabilitation training (RT) for the treatment of post-stroke limb spasticity (PSLS).

**Methods:**

We will search Cochrane Library, MEDILINE, EMBASE, CINAHL, AMED, PsycINFO, WOS, Scopus, OpenGrey, and 4 Chinese databases from inception to the present without language restrictions. We will only consider randomized controlled trial on assessing the effectiveness and safety of NMES combined with RT for the treatment of PSLS. All included randomized controlled trials will be assessed using Cochrane risk of bias tool. Two researchers will independently perform study selection, risk of bias assessment, and data extraction, respectively. Any disagreements will be solved by a third researcher through discussion.

**Results:**

Primary outcome is limb spasticity status. Secondary outcomes comprise of limb function, quality of life, and adverse events.

**Conclusion:**

This study will summarize the latest evidence of NMES combined with RT for the treatment of patients with PSLS.

**Systematic review registration:**

PROSPERO CRD42019138900.

## 1 Introduction

Post-stroke limb spasticity (PSLS) is a very common complication among stroke survivors.^[1–3]^ This disorder often involves in voluntary movement, which greatly limit the mobility and functional ability of patients with PSLS, and can decrease their quality of life.^[4–7]^ It has been estimated that the prevalence of PSLS is 19% to 42.6%,^[7,8]^ and its disabling spasticity varies from 2% to 13%.^[9]^ To treat patients with PSLS, a number of different managements such as neuromuscular electrical stimulation (NMES), rehabilitation training (RT), local botulinum toxin injection, and surgical interventions have been commonly utilized in clinic.^[3,10–16]^ However, the efficacy of those single treatments is still limited. Thus, it is very important to use combined approaches to treat this disorder, such as NMES combined with RT. Although several clinical studies have reported that the efficacy of NMES plus RT is encouraging,^[3,12,17–20]^ the conclusion is still unclear. Therefore, this systematic review will systematically assess the efficacy and safety of NMES combined RT for patients with PSLS.

## 2 Methods

### 2.1 Dissemination and ethics

The results of this study are expected to be published at peer-reviewed journals. We will not use individual data, thus, no ethic approval is needed.

### 2.2 Study registration

This study protocol has been registered with PROSPERO CRD42019138900. Its report has followed the Cochrane Handbook for Systematic Reviews of Interventions and the Preferred Reporting Items for Systematic Reviews and Meta-Analysis Protocol (PRISMA-P) statement guidelines.^[21]^

### 2.3 Inclusion criteria for study selection

#### 2.3.1 Types of studies

This study will only include randomized controlled trials (RCTs) of NMES combined with RT for treatment of PSLS. All other studies will be excluded, including non-clinical trial, and non-RCTs.

#### 2.3.2 Types of participants

Patients with PSLS regardless of race, sex, and age will be included. However, we will exclude patients with severe congestive heart failure or severe chronic obstructive lung disease.

#### 2.3.3 Types of interventions

In the experimental group, all patients have received NMES combined with RT.

In the control group, all patients have received any treatments, except NMES and RT.

#### 2.3.4 Type of outcome measurements

Primary outcome includes limb spasticity status, which can be assessed by any scales, such as Modified Ashworth Scale, or others. Secondary outcomes consist of limb function, which can be measured by Disability Assessment Scale or other tools; and quality of life, which can be evaluated by Assessment of Quality of Life, or any other scales; as well as the expected or unexpected adverse events.

### 2.4 Search methods for the identification of studies

#### 2.4.1 Electronic database searches

The following databases of Cochrane Library, MEDILINE, EMBASE, PsycINFO, WOS, Scopus, OpenGrey, Cumulative Index to Nursing and Allied Health Literature, Allied and Complementary Medicine Database, Chinese Biomedical Literature Database, China National Knowledge Infrastructure, VIP Information, and WANFANG Databases will be searched from inception to the present without language restrictions. All RCTs of NMES combined with RT for the treatment of PSLS will be included. The search strategy for Cochrane Library is demonstrated in Table 1. Identified strategies of other electronic databases will also be adapted.

**Table 1:**
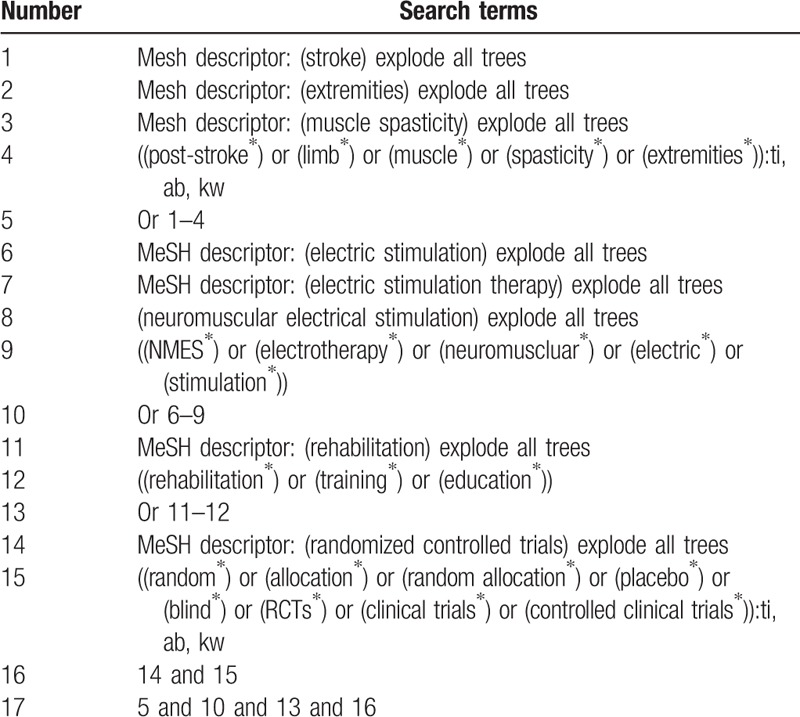
Search strategy for Cochrane Library database.

#### 2.4.2 Search for other resources

Aside from all electronic databases, clinical registry, and reference list of included studies will also be searched.

### 2.5 Data collection and analysis

#### 2.5.1 Study selection

**Figure 1:**
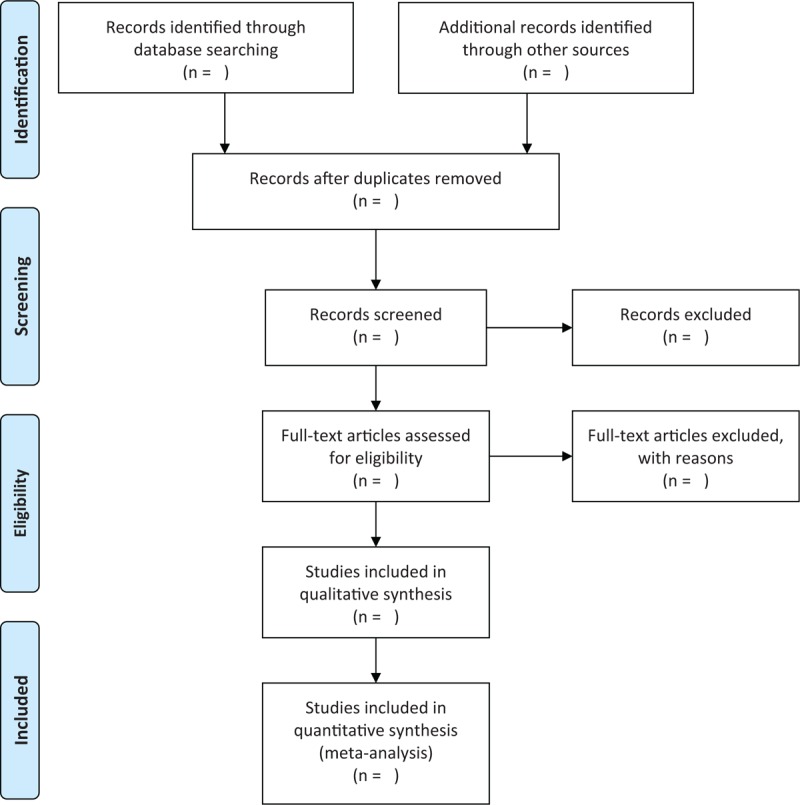
Flow diagram of study selection.

Two researchers will independently assess titles and abstracts of identified records for eligibility. All process of study selection follows the inclusion criteria and exclusion criteria. If the records fit the inclusion criteria, full-texts will be further read to check its eligibility. Any disagreements between 2 researchers will be solved by discussion with a third researcher. The selection procedure will be abided and presented in the PRISMA flow chart in Fig. 1.

#### 2.5.2 Data collection and management

Details of all eligible studies will be extracted and summarized using a data extraction form. Two researchers will independently collect such information. Any diverges will be resolved by a third researcher through discussion. The following extracted data consist of study characteristics, patient characteristics, study design, study methods, treatment details, outcome measurements, and safety.

#### 2.5.3 Risk of bias assessment

We will utilize Cochrane Handbook for Systematic Reviews of Interventions tool to evaluate risk of bias for all eligible RCTs. It covers 7 domains, and each domain is further judged as 3 levels of high, unclear, or low risk of bias. Two researchers will independently carry out risk of bias assessment, respectively. Any divergences will be resolved by a third researcher.

#### 2.5.4 Measurement of treatment effect

For each eligible RCTs with enumeration data, we will calculate it as risk ratio and 95% confidence intervals, while as for continuous data, we will calculate it as mean difference or standardized mean difference with 95% confidence intervals.

#### 2.5.5 Dealing with missing data

If the essential information is missing or insufficient, the original authors will be contacted to obtain that information. We will analyze available data alone if we cannot obtain that information.

#### 2.5.6 Assessment of heterogeneity

Heterogeneity will be checked by *I*^2^ test. *I*^2^ ≤ 50% means reasonable heterogeneity. *I*^2^ > 50% means significant heterogeneity. Under such situation, subgroup analysis will be carried out.

#### 2.5.7 Data synthesis

RevMan 5.3 software (London, UK) will be used for statistical analysis. If heterogeneity is reasonable among eligible RCTs (*I*^2^ ≤ 50%), a fixed-effects model will be pooled, and a meta-analysis will be performed. Otherwise, if the heterogeneity is reasonable among eligible RCTs (*I*^2^ > 50%), a random-effect model will be utilized, and meta-analysis will be performed according to the results of subgroup analysis. A narrative summary will be reported instead of meta-analysis if there is still significant heterogeneity after subgroup analysis.

#### 2.5.8 Publication bias

Funnel plot and Egger regression will be used for check publication bias if >10 studies are entered.^[22]^

#### 2.5.9 Subgroup analysis

We will perform subgroup analysis in accordance with different interventions, controls, and outcomes.

#### 2.5.10 Sensitivity analysis

We will carry out sensitivity analysis to check robustness and satiability of pooled outcome results by removing low quality RCTs.

## 3 Discussion

This study aims to systematically investigate the efficacy and safety of NMES and RT for treating PSLS. To our best knowledge, this systematic review firstly assesses the efficacy and safety of NMES plus RT for treating PSLS. The results of this study summarize the latest evidence of NMES and RT for the treatment of patients with PSLS. Its findings may either benefit patients and clinicians, or help health policy-makers.

## Author contributions

**Conceptualization:** Ya-long He, Yan Gao, Bai-ya Fan.

**Data curation:** Ya-long He, Yan Gao, Bai-ya Fan.

**Formal analysis:** Ya-long He, Yan Gao.

**Funding acquisition:** Yan Gao.

**Investigation:** Yan Gao.

**Methodology:** Ya-long He, Bai-ya Fan.

**Project administration:** Yan Gao.

**Resources:** Ya-long He, Bai-ya Fan.

**Software:** Ya-long He, Bai-ya Fan.

**Supervision:** Yan Gao.

**Validation:** Ya-long He, Yan Gao, Bai-ya Fan.

**Visualization:** Ya-long He, Yan Gao, Bai-ya Fan.

**Writing – original draft:** Ya-long He, Yan Gao, Bai-ya Fan.

**Writing – review & editing:** Ya-long He, Yan Gao, Bai-ya Fan.
